# Recombinant human complement component C2 produced in a human cell line restores the classical complement pathway activity *in-vitro: *an alternative treatment for C2 deficiency diseases

**DOI:** 10.1186/1471-2172-11-43

**Published:** 2010-08-20

**Authors:** Paolo GV Martini, Lynette C Cook, Scott Alderucci, Angela W Norton, Dianna M Lundberg, Susan M Fish, Knut Langsetmo, Göran Jönsson, Christian Lood, Birgitta Gullstrand, Kate J Zaleski, Nancy Savioli, Jason Lottherand, Charles Bedard, John Gill, Michael F Concino, Michael W Heartlein, Lennart Truedsson, Jan L Powell, Arthur O Tzianabos

**Affiliations:** 1 Department of Protein Expression and Purification Research, Shire Human Genetic Therapies Inc., 700 Main Street, Cambridge, MA 02139, USA; 2 Department of Infectious Diseases, Lund University Hospital, Klinikgatan 3, Lund 221 85, Sweden; 3 Department of Laboratory Medicine, Section of Microbiology, Immunology and Glycobiology, Lund University, Solvegatan 23, Lund 223 62, Sweden; 4 Department of Physiology, Shire Human Genetic Therapies Inc., 700 Main Street, Cambridge, MA 02139, USA; 5 Department of Cell Culture Process Development, Shire Human Genetic Therapies Inc., 700 Main Street, Cambridge, MA 02139, USA; 6 Department of Discovery, Shire Human Genetic Therapies Inc., 700 Main Street, Cambridge, MA 02139, USA

## Abstract

**Background:**

Complement C2 deficiency is the most common genetically determined complete complement deficiency and is associated with a number of diseases. Most prominent are the associations with recurrent serious infections in young children and the development of systemic lupus erythematosus (SLE) in adults. The links with these diseases reflect the important role complement C2 plays in both innate immunity and immune tolerance. Infusions with normal fresh frozen plasma for the treatment of associated disease have demonstrated therapeutic effects but so far protein replacement therapy has not been evaluated.

**Results:**

Human complement C2 was cloned and expressed in a mammalian cell line. The purity of recombinant human C2 (rhC2) was greater than 95% and it was characterized for stability and activity. It was sensitive to C1s cleavage and restored classical complement pathway activity in C2-deficient serum both in a complement activation ELISA and a hemolytic assay. Furthermore, rhC2 could increase C3 fragment deposition on the human pathogen *Streptococcus pneumoniae *in C2-deficient serum to levels equal to those with normal serum.

**Conclusions:**

Taken together these data suggest that recombinant human C2 can restore classical complement pathway activity and may serve as a potential therapeutic for recurring bacterial infections or SLE in C2-deficient patients.

## Background

Our understanding of the role of complement in human disease is the result of numerous studies in recent years focused on complement's mechanism of action. This has resulted in achieving important information on the role of complement as a major mediator and effector mechanism in diseases of immune and non-immune pathogenesis. Complement is not only important for protection against microorganisms, but also contributes to the pathophysiology of a number of autoimmune diseases.

Progress regarding the biological role of complement has been made by studying disease associations in patients with inherited complement protein deficiencies [1]. Genetic deficiencies of complement components are a common denominator of immune and infectious diseases. Deficiencies of complement components of the classical activation pathway, C1, C2 and C4, all lead to increased susceptibility to bacterial infections [2] and increased risk of developing autoimmune disease, particularly systemic lupus erythematosus (SLE) [3]. The complement system consists of more than 30 soluble and membrane proteins and constitutes an important mediator of host defense against foreign pathogens. Complement component C2 functions as a key regulator in the early activation phase of the classical pathway and participates in the formation of the classical pathway C3 convertase C4b2a [4]. C2 is also a critical component of the lectin pathway. Specifically, when mannose-binding lectin (MBL) or ficolins in complex with MBL-associated serine protease (MASP) molecules bind to relevant carbohydrate molecules, this leads to activation of MASP-2 which then may cleave both C2 and C4 thereby forming the same C3 convertase as in classical pathway activation [5]. Thus, C2 is an important component of both the classical and the lectin pathways of complement activation and is involved in first line defense against microbial infection that is essential for detection and clearance of the invading pathogens [6].

Complement C2 deficiency is the most common genetically determined complete complement deficiency with a prevalence estimated to be approximately 1:20,000 in individuals of Caucasian ancestry [3], making it a clinically important immune deficiency [7]. The deficiency is, in the majority of cases, caused by homozygosity for C2 genes having deletions in exon 6, resulting in complete absence of C2, or in some cases caused by other C2 gene mutations [8,9]

The alternative activation pathway, which is C3 dependent, is generally intact in C2 deficiency and can trigger formation of the membrane attack complex (MAC) independently of C2 [4]. However, in the absence of C2, C3 is, in many situations, not efficiently cleaved resulting in a limited deposition of C3 fragments on immune complexes and on the surface of apoptotic cells. Circulating apoptotic cells become a source of self antigen for auto-antibodies that participate in the formation of immune complexes. The immune complexes are deposited throughout the body, potentially causing localized inflammatory reactions in joints and kidneys, and ultimately leading to renal disease from chronic activation of the complement system [10].

In this study, we considered C2 replacement as a therapeutic target to explore the feasibility of restoring the complement pathway in cases of C2 deficiency. It has been previously proposed that purified human C2 could restore classical and lectin complement pathways and hemolytic activity *ex-vivo *in serum collected from C2-deficient patients [11]. Two case histories have been described where regular infusions of fresh frozen plasma were beneficial in patients with C2-deficiency and SLE; this benefit was ascribed to the C2 contained in the fresh frozen plasma [12,13]. Therefore, we hypothesized that recombinant human complement component C2 (rhC2) could restore complement activity in serum from C2-deficient patients. To test this hypothesis we have expressed and purified rhC2 and assessed complement activation *in vitro*. To our knowledge this is the first report of proof of concept for treating C2 deficiency with recombinant human C2.

## Results

### Protein Cloning and Expression

**Figure 1:**
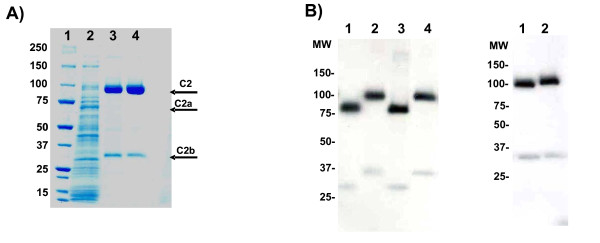
**Expression, purification and post-translational modification of rhC2**. A) Coomassie stained SDS-PAGE gel of rhC2 at different stages of the purification process. Each lane contains 0.5 μg protein: Lanes: 1. Molecular weight marker, 2. conditioned media, 3. Sepharose pool, 4. purified C2. B) rhC2 glycodigestion with Peptide N-glycosidase (PNGase-F) shows removal of carbohydrates from the protein backbone. Lanes: 1. rhC2 + 1 hour PNGase-F, 2. rhC2 untreated 1 hour, 3. rhC2 + overnight PNGase-F, 4. rhC2 untreated overnight. C) Neuramidase digestion of rhC2 reveals sialic acid removal after digestion of the protein. Lanes: 1. rhC2 + 1 hour Neuramidase, 2. rhC2 untreated.

**Figure 2:**
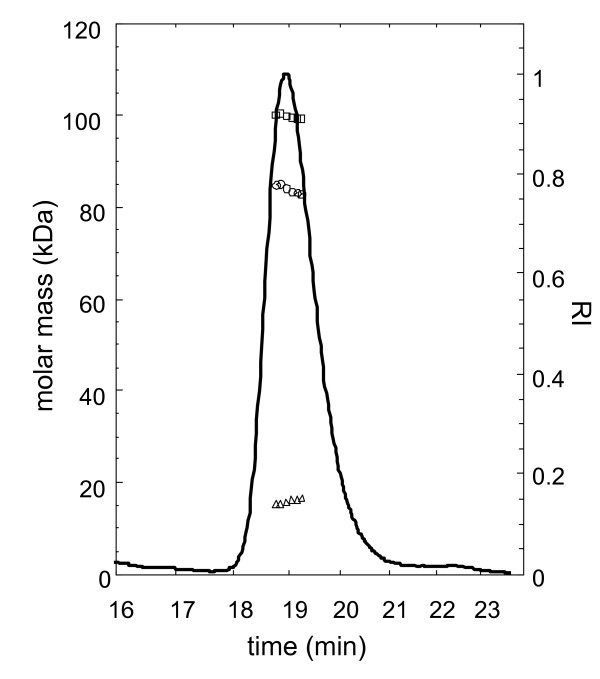
**Analysis of rhC2 glycosylation by SEC-MALS**. rhC2 was eluted from a Tosoh Super SW3000 size exclusion column and monitored by normalized differential refractive index (─) and absorbance at 280 nm versus time. Molar mass was analyzed by multi angle light scattering following the total mass by differential refractive index (□), and protein mass by absorbance at 280 nm (Ο). The molar mass contributed by glycosylation is plotted as the difference between the total mass and the mass of the protein (**Δ**). The average molar mass determined for the total is 99.8 kDa, the protein 84 kDa, and the carbohydrate 15.8 kDa.

**Figure 3:**
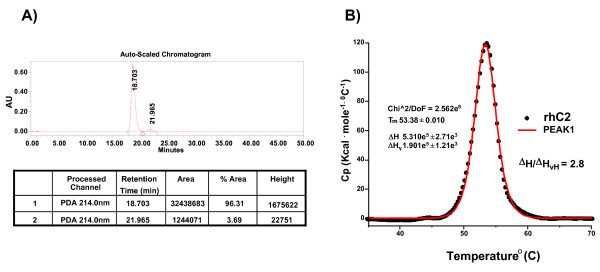
**Biochemical analysis of rhC2**. A) Chromatogram representing the purity of rhC2 by size exclusion chromatography: purity was calculated to be ~96%. The % area (% purity) was quantified using Exponential Skim Integration of the Empower software. B) Differential scanning calorimetry: 400 μg/ml of protein were loaded in the calorimetry cell and heated at a rate of 60°C/hour. The excess heat capacity *vs*. temperature shows a single unfolding transition with a midpoint at (T_m_) of 53.4°C.

The rhC2 was highly expressed in human cells and shown to be purified very efficiently by a 2-step column chromatography process (Figure 1A) based on a modification and improvement of a procedure previously described [14,15] (some cleaved products of rhC2, C2a and C2b, are present in the conditioned media as a result of endogenous proteases produced by the cells). After purification, a major band, representing rhC2, is visible by Coomassie staining as well as a smaller band, C2b, the inactive part of the cleaved C2, which co-purifies with the full length protein and constitutes a minor impurity. C2a is completely removed during the purification process. An early report on C2 analysis indicates that the mature protein has eight glycosylation sites [16]. To determine post-translational modification of rhC2 we performed a glycodigestion with Peptide N-glycosidase and show that rhC2 is glycosylated when produced in human cells (Figure 1B). Neuramidase digestion of the protein also demonstrated the presence of sialic acid on rhC2 (Figure 1C), which may result in greater stability of the protein in serum [17]. By multi angle static light scattering (MALS) the weight average molar mass (M_w_) of the full length rhC2 including glycosylation was determined to be 99,800 g/mol (Figure 2). The number average molar mass (M_n_) can also be determined by SEC-MALS, with the ratio M_w_/M_n _providing a measure of polydispersity. The polydispersity index of rhC2 is 1.00054, indicating narrow mass distribution. The line in figure 2 represents the differential refractive index (dRI) versus elution time, indicating the elution of rhC2. The data points in figure 2 are the molar mass determined from the light scattering intensity using the concentration measured by dRI for the glycoprotein complex, and the absorbance at 280 nm for the protein component. The molar mass of the protein component was 84,000 g/mol and the difference in the two masses was the weight of the modifiers (glycosylation) which was 15,800 g/mol. The 84,000 g/mol mass of the protein without any modifiers was consistent with the expected molecular weight calculated from the protein sequence of ~ 82000 kDa. The purity of rhC2 determined by size exclusion chromatography (SEC) was greater than 95% (Figure 3A) with minor impurities consisting mainly of C2b, characterized also by MALDI-TOF analysis (data not shown).

Differential scanning calorimetry (DSC) showed a single temperature induced unfolding transition with a temperature midpoint (T_m_) of 53.4°C (Figure 3B). The ratio of the calorimetric enthalpy (ΔH_cal_) to the van't Hoff enthalpy (ΔH_vH_) was 2.8, consistent with the three structural domains of rhC2 unfolding in a single transition. Surveys comparing T_m _and ΔH at various pH values and salt concentrations indicate that rhC2 is most stable at pH 6.5 and 150 mM NaCl (data not shown).

### rhC2 is cleaved by C1s and is active in C2 depleted serum

**Figure 4:**
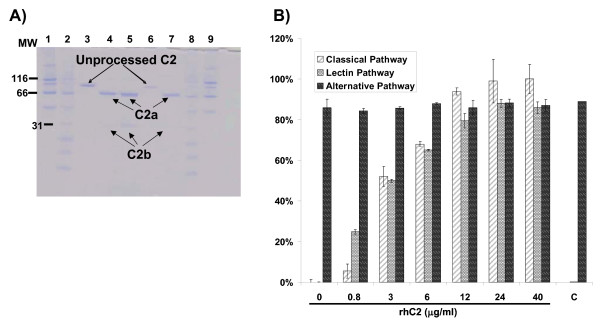
**rhC2 characterization**. A) Cleavage of rhC2 by complement protease C1s. Lanes: 1. High molecular weight marker, 2. Low molecular weight marker, 3. rhC2 undigested, 4. rhC2 (25 μg/ml) digested with C1s, 5. rhC2 (50 μg/ml) digested with C1s, 6. plasma purified C2, 7. plasma purified C2 (25 μg/ml) digested with C1s, 8. Low molecular weight marker, 9. High molecular weight marker. B) Reconstitution of complement activity as measured by complement activation ELISA by rhC2 in C2 affinity depleted human serum. Normal human serum was used as standard (100%). Dose response of rhC2 in serum shows activation of the classical pathway at a concentration as low as 3 μg/ml. As negative control, an irrelevant protein (C) was run to confirm specificity of the assay to rhC2. Data represent the mean ± SD of three independent experiments.

As a first step in the activation of the classical complement pathway, complement protein C1q binds to antibodies and triggers the activation of C1r and C1s. Activated complement factor C1s cleaves C2 and C4 so they can form the C3 convertase thereby triggering the terminal portion of the complement cascade [15]. Therefore, in order for C2 to be fully functional, it must first be cleaved by C1s. Cleavage of C2 results in release of C2a (70 kDa) which in association with C4b serves as the C3 convertase. A smaller protein, C2b (30 kDa), is also released following cleavage by C1s. To determine that rhC2 exhibited the correct functional conformation, purified protein was incubated with purified C1s and the resultant proteins visualized by SDS-PAGE and Coomassie staining (Figure 4A). Results from these studies show that rhC2 is cleaved by C1s, releasing C2a and C2b proteins of the correct molecular weight. C2 purified from human plasma was included as a positive control. These data suggest that rhC2 forms the correct functional conformation to activate the classical complement pathway.

To determine whether rhC2 was able to restore complement activation through the classical pathway, purified recombinant protein was diluted in affinity depleted C2-deficient serum (0 - 40 μg/ml) and tested using the Total Complement (3-Pathway) ELISA. The 3-pathway ELISA measures the ability of complement proteins in a given serum sample to activate the classical, lectin or alternative pathway based on specific antigens used to trigger each pathway of the cascade. Results show the specific reconstitution of the classical and lectin complement pathways when rhC2 was added to the C2-depleted serum but these pathways were not activated in the absence of rhC2 (Figure 4B). An rhC2 concentration of 3 μg/ml restored > 50% classical pathway activity, with 100% activation at 12 μg/ml. As expected, the alternative pathway, which is independent of C2, was activated by C2-depleted serum. An irrelevant protein (a transferrin fusion protein) with similar MW was used as negative control and did not activate the complement pathway.

### C3 deposition on bacterial pathogen Streptococcus pneumoniae

**Figure 5:**
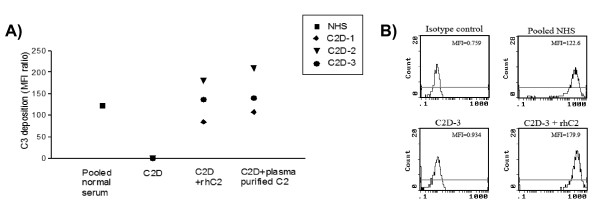
**Effect of rhC2 on deposition of C3 fragment on the surface of *Streptococcus pneumoniae *analyzed by flow cytometry**. The bacteria were incubated with C2 deficient (C2D) serum from patients, C2D serum with (25 μg/ml) rhC2 or plasma purified C2. Pooled normal human serum (NHS) was used as control. A) Results with 3 different C2D sera expressed as mean fluorescence index (MFI) ratio between the recorded fluorescence with anti-C3d antibody and the fluorescence obtained with the isotype control antibody. B) Flow cytometry plots showing MFI of C3 fragment deposition with one C2D serum (C2D-3) before and after restitution with rhC2.

Susceptibility to encapsulated bacterial pathogens such as *Streptococcus pneumoniae*, *Neisseria meningitidis *and *Haemophilus influenzae *is a hallmark of C2 deficiency, particularly in young children (≤ 2 years) (2). The classical complement pathway plays a key role in the clearance of these pathogens through antibody and non-antibody-mediated mechanisms. Complement activation by antibodies to encapsulated bacterial pathogens results in the deposition of C3b on the surface of the bacterium in association with C3 cleavage by the C4b2a complex. In the absence of C2, only C4b is bound to the bacterial cells as a result of the classical pathway activation initiated by the specific antibodies. The addition of 25 μg/ml rhC2 or C2 purified from plasma to C2-deficient serum increased the C3d deposition on *Streptococcus pneumoniae *to levels similar to those using a pool of normal serum (Figure 5A). Thus, we could demonstrate that addition of C2 to C2-deficient serum highly increased the C3 fragment deposition on the bacteria. Figure 5B shows flow cytometry plot of C3 fragment deposition using a serum sample from a C2-deficient patient serum before and after restitution with rhC2

### Reconstitution of classical pathway activity in serum from C2-deficient patients

Considering rhC2 as a therapeutic alternative for C2 deficiency-associated diseases, it was important to test the concept in serum collected from human patients homozygous for C2 deficiency. We have used the 3-pathway ELISA that measures complement cascade activation as well as a hemolytic assay that measures classical pathway activity by determining the ability of serum complement proteins to bind to antibody-coated sheep red blood cells. Successful activation of the classical pathway results in formation of the MAC and red blood cell lysis. Serum from six different C2-deficient patients was reconstituted with rhC2 and tested in the 3-pathway ELISA according to the manufacturer's instructions and three C2-deficient sera were also analyzed by the hemolytic assay [18]. In addition, rhC2 was titrated and tested in C2-deficient serum from patients in the 3-pathway ELISA to investigate the minimum amount of protein needed to restore classical pathway activity to levels similar to those in pooled normal human serum.

**Figure 6:**
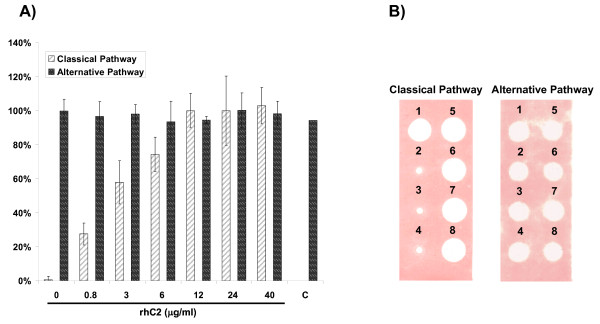
**rhC2 restores classical pathway complement activity in C2-deficient patient serum**. A) Representative titration of rhC2 in serum from a C2-deficient patient and measurement of complement pathway activity by complement activation ELISA. "C' denotes inclusion of an irrelevant protein used to confirm specificity of the assay to rhC2. Data represent the mean ± SD of three independent experiments. B) rhC2 restores classical pathway hemolytic activity in C2-deficient patient sera. When rhC2 (25 μg/ml) was added to the sera from 3 different patients, complement activation was triggered and hemolysis observed. Samples no. 1 Normal human serum, 2 - 4 C2-deficient patient sera, 5-7 the C2-deficient patient sera restituted with rhC2 (25 μg/ml), 8 C2-deficient serum restituted with plasma purified C2 (25 μg/ml). Alternative pathway of hemolysis as a control is also shown.

Results from the 3-pathway ELISA and hemolytic assay show that rhC2 restores the capacity of C2-deficient patient serum for classical pathway activation enabling the formation of the MAC (Figures 6A and 6B). Titration of rhC2 revealed that a concentration of 3.0 - 6.0 μg/ml was sufficient to activate the classical complement pathway to ≥70% activity of normal human serum in different C2-deficient patient sera (Figure 6A). As a reference, normal human serum from healthy individuals contains 16 - 40 μg/ml of circulating C2. No activation of the lectin pathway was observed in most of these patient sera due to low serum levels of MBL (Table 1).

**Table 1:** Restitution of Classical (CP), Lectin (LP) and Alternative pathway (AP) activation in serum from 6 C2-deficient patients by addition of rhC2

**Restituted serum **^**a**^ Patient no.	CP	**LP**^**c**^	AP
1	+++**^b^**	±	+++
2	+++	-	+++
3	+++	±	+++
4	+++	+	+++
5	+++	++	+++
6	+++	-	+++

^a^rhC2 was added to C2-deficient serum, final concentration 25 μg/ml.

^b^Complement activation tested by ELISA. +++ = 100% activation, ++ = 50%, + = 20%, - = no activation.

^c^LP activity reflects the MBL concentration governed by MBL genotype. Patient 5 is homozygous for the wild-type structural gene variant (genotype A/A), while the other patients are heterozygous for this allele.

## Discussion

In this study we provide proof of concept that rhC2 produced in human cells could be considered as an alternative treatment for C2-deficient diseases. This concept was originally formulated by Ruddy *et al *[11], where in an *ex-vivo *experiment they reconstituted hemolytic activity of C2-deficient serum collected from a patient by adding C2 purified from normal human serum. We have expanded this concept further and have demonstrated that rhC2 can be expressed in high quantities from human cells. We have prepared highly purified rhC2, shown that it undergoes post-translational modification and that it is very stable. rhC2 is cleaved *in-vitro *by C1s protease generating the active subunit of C2 (C2a) necessary to form the C3 convertase C4b2a and more importantly, it restores the classical and lectin pathways in C2-depleted and C2-deficient serum at different concentrations. The fact that we were able to show normalization of complement activation at concentrations of rhC2 (3-6 μg/mL) that are lower than those typically observed in normal human serum (16-40 μg/mL) is in accordance with the reports that the heterozygous C2-deficiency state is not a risk factor for disease [3], and confirms that the rhC2 is fully functional.

To our knowledge this is the first report to date describing the production, purification and functional activity of rhC2 produced in human cells. In previous reports C2 was either from plasma of human or animal origin or from mouse L-cells or monkey COS cells [14,19-23]. The advantage of expressing C2 in human cells is that it may result in a more naturally glycosylated protein; most likely very similar or identical to the human C2 secreted in normal individuals, which is, in our opinion, an important consideration for the development of a therapeutic protein. We have also shown that rhC2 restores the classical pathway activation *in-vitro *in C2-deficient serum obtained from six different patients. The use of C2-deficient human serum represents the proof of concept for therapeutic use of rhC2 as an alternative therapy for C2 deficiency-associated diseases. Restoration of the classical pathway in human C2-deficient serum with rhC2 could be an important step, for instance, in treating C2-deficient SLE patients refractory to current therapies. Interestingly, it has been previously reported in two patients that infusion of normal fresh frozen plasma as a source of complement C2 provided therapeutic benefit [12,13]. Immunological studies performed in one of these patients revealed that these infusions led to increased serum C2 levels, increased hemolytic activity, decreased immune precipitation and decreased circulating immune complexes [13,24]. Although it has been argued that the observed clinical effects of fresh frozen plasma were a result of multiple components in the plasma (Factor B, immunoglobulin, MBL) other than C2, it has been demonstrated that treatment with IgG only in the amount corresponding to one course of plasma infusion [24] did not have any beneficial effect, suggesting that the key player in promoting complement activity through the classical pathway, was C2 alone. In addition, infusion of rhC2 may decrease the risk of developing adverse reactions compared to plasma infusion as plasma contains many proteins that may trigger different reactions in patients.

The therapeutic use of rhC2 could also be considered in the case of C2-deficient individuals with recurrent or invasive bacterial infections. It has been shown that C2 deficiency is associated with increased susceptibility to recurrent infections by encapsulated bacterial pathogens such as *Streptococcus pneumoniae *[7,25,26]. Complement activation resulting in opsonization of the bacteria with C3 fragments is an important defense mechanism which is impaired in C2 deficiency [27]. Therefore, we could envision rhC2 as an adjunct therapy to antibiotics, with the rhC2 facilitating phagocyte-mediated clearance of the pathogen by enabling full complement cascade activation.

Blood protein replacement therapy is well established in some clinical situations, for example, intravenous immunoglobulin in patients with antibody deficiencies or coagulation factors in the hemophilias and specific lysosomal hydrolases in lysosomal storage diseases [28]. Drotrecogin alpha, a recombinant form of activated protein C, is approved for use in sepsis and Diffuse Intravascular Coagulation. The only complement replacement therapy already established is in hereditary angioedema caused by C1-inhibitor deficiency. Plasma-derived C1-inhibitor is shown to be an efficient treatment [29] and recombinant C1-inhibitor is under clinical evaluation [30]. Another complement protein that has been tried as replacement therapy is MBL in patients with MBL deficiency and clinical disease, related to the deficiency. Plasma-derived MBL has been used [31] and recently recombinant MBL was shown to successfully restore the MBL activation pathway *in vivo *[32]. Thus, all together these examples suggest that C2 replacement therapy could be considered for further evaluation of possible therapeutic use in humans. Recombinant human proteins offer many benefits over purified products from pooled human plasma, such as a lower risk of transmission of infectious diseases and higher batch-to-batch consistency [33]. Therefore, it was important to show that rhC2 can substitute for endogenously produced C2 in its immune and anti-infective effects. The present report shows that rhC2 is fully functional *in vitro *and can be considered for protein replacement therapy in C2 deficiency-associated diseases.

## Conclusions

We have demonstrated that a biochemically well characterized recombinant human complement component C2 produced in a human cell line restores classical complement pathway activation *in vitro*, in serum from C2-deficient patients. Establishing efficacy in specific animal models for SLE and infectious diseases will further validate this concept; although the present study provides the first evidence for protein replacement therapy as a treatment of diseases related to C2 deficiency.

## Methods

### Cell culture and protein expression

Complement component C2 (NM_000063) was cloned from a liver cDNA library (Invitrogen, Carlsbad, CA), and inserted into a plasmid carrying a collagen promoter (pX804) or into pCep4 (Invitrogen, Carlsbad, CA). HT1080 cells (CCL-121, ATCC, Manassas, VA) were grown and maintained in CD media (50% CD-CHO and 50% CD-293) (Invitrogen, Carlsbad, CA) at 37°C in a 5% CO_2 _incubator. Stable transfections were carried out using electroporation (450 V with 250 μF of capacitance and 30 μg of plasmid DNA suspended in 750 μl 1× PBS). 293-F cells (Invitrogen, Carlsbad, CA) were grown in Freestyle 293 media (Invitrogen, Carlsbad, CA) containing GlutaMAX. Cells were transiently transfected using a polyethylenimine (25 kD, Polysciences, Warrington, PA) to DNA ratio of 3:1 and were supplemented with 2.5 mg/ml Primatone (MP Biomedicals, Solon, OH) at 24 hours. Seventy-two hours after transfection conditioned media enriched in rhC2 was harvested from a 25 liter wave bioreactor (GE Healthcare, Piscataway, NJ) and then processed for purification.

### Protein purification and biochemical characterization

A two-step purification process was developed for rhC2. First a SP Sepharose Fast Flow resin column (GE Healthcare, Piscataway, NJ) was used to capture the protein from harvest medium. The binding capacity of the SP resin was experimentally determined by ELISA to be 0.37 mg rhC2 per ml of SP resin. Protein was then eluted from the column and samples from collected fractions were analyzed by BCA Protein Assay Kit, (Pierce, Rockford, IL) to determine protein concentration and visualized by Coomassie staining (GelCode Blue Stain Reagent, Pierce, Rockford, IL) of 8-16% tris-glycine SDS-PAGE gels under reducing conditions (Invitrogen, Carlsbad, CA). The fractions containing full length rhC2 (102 kDa) were pooled and further purified over a Heparin Sepharose Fast Flow resin column (GE Healthcare, Piscataway, NJ). A binding capacity of 1 mg of total protein by BCA assay per ml of Heparin resin was used. Eluted fractions were assessed as described above and those containing rhC2 were pooled, concentrated and buffer-exchanged into storage buffer (10× PBS diluted to 1× in water and adjusted to pH 7). Forty five liters of harvest media from the WAVE bioreactor yielded 228 mg of purified rhC2. The resultant rhC2 protein was confirmed by N-terminal sequencing (Mayo Proteomics Research Center, Mayo Clinic College of Medicine in Rochester, MN) and was assessed for purity using size exclusion chromatography. Purified rhC2 (5 μg) was separated on a size exclusion chromatography (SEC) column (Tosoh SuperSW 3000, 10,000-500,000 Da, Tosoh, Montgomery, PA) with 25 mM sodium phosphate, pH 6.5 and 500 mM sodium chloride in MilliQ water as the mobile phase with a flow rate of 0.15 ml/min. The chromatogram was quantified using Exponential Skim Integration of the Empower software (Waters, Milford, MA). Using this procedure, the resultant rhC2 was determined to be 96% pure. The molecular weight and hydrodynamic radius of the major peak seen on size exclusion chromatography for rhC2 was analyzed using a multi angle static and dynamic light scattering system from Wyatt Technologies (Santa Barbara, CA) in conjunction with size exclusion chromatography method described above with the following modifications: the pH of the light scattering mobile phase buffer was increased to pH 7.0, the flow rate was increased to 0.3 mL/min, and 10 μg of rhC2 diluted (1:10) in light scattering mobile phase buffer.

Glycodigestion was performed to determine the presence of carbohydrates and sialic acid. Two micrograms of rhC2 was used for glycodigestion, for carbohydrate determination the protein was incubated for 1 hour and overnight at 37°C with Peptide N-glycosidase in reaction buffer (New England Biolabs, Beverly, MA). For sialic acid determination, rhC2 was incubated for 1 hour at 37°C with neuraminidase in reaction buffer (New England Biolabs, Beverly, MA). SDS-PAGE was performed with 250 ng of sample digested with above enzymes, loaded on 8-16% gel and visualized by Western blot. Thermal stability of rhC2 was assessed with differential scanning calorimetry using a MicroCal VP-DSC (GE Healthcare, Piscataway, NJ) with 400 μl buffer (25 mM sodium phosphate, 500 mM sodium chloride) per buffer/sample pairs in 96 deep well plates loaded in the CapVP-DSC autoloader. Data were collected with the calorimeter scanning from 10 to 100°C at a rate of 60°C/hour.

A sandwich ELISA was developed to allow specific quantitation of rhC2 during purification. The wells of a 96-well Nunc Immuno plate (Nalge Nunc International, Rochester, NY) were coated with 100 μl of 1 μg/ml anti-human C2 goat polyclonal antibody (R&D systems, Minneapolis, MN) in 50 mM sodium bicarbonate, pH 9.6. The plate was sealed and incubated overnight at RT. The next morning the wells were washed 3 times with ELISA Wash Buffer (0.1% Tween-20 in PBS), then 300 μl of Blocking Buffer (PBS, 0.05% Tween-20, 2% BSA) were added to each well and the plate was incubated for 1 hour at 37°C. Following the incubation, blocking buffer was removed, 100 μl volume of samples and standards (pre-diluted in blocking buffer) were added to the plate, and the plate was incubated for 1 hour at 37°C. Following incubation, the plate was washed as described above, the secondary antibody, a mouse anti-human C2 monoclonal antibody (Abcam, Cambridge, MA), was added to all wells at a final concentration of 1 μg/mL (100 μl per well) and the plate was incubated for 1 hour at 37°C then washed. After washing, 100 μl of 100 ng/ml of HRP-conjugated anti-mouse antibody (Promega, Madison, WI) was added to each well and the plate was incubated for 1 hour at 37°C then washed. Finally, 100 μl of the complete Peroxidase EIA Substrate Kit (BioRad, Hercules, CA) was added to each well and the plate incubated for 20 min at 37°C. The reaction was stopped by adding 100 μl of 2 N H_2_SO_4 _to each well and the plate was read at 450 nm on a Molecular Devices plate reader equipped with SoftMax Pro software. The standard curve had a dynamic range from 200 ng/ml to 3 ng/ml prepared by serially diluting C2 purified from human serum (R&D systems, Minneapolis, MN).

### Cleavage of rhC2 by C1s

rhC2 and purified C2 from human plasma at a concentration of 25 μg/ml and 50 μg/ml, respectively were incubated with active C1s for 1 h at 37°C in veronal buffered saline with 0.15 mM Ca^2+ ^and 0.5 mM Mg^2+ ^(VBSCaMg) at a concentration of 70 μg/ml to induce complete cleavage of C2. C1s was produced and purified as described by Arlaud G.J. *et al*, [34]. The proteins were then analyzed by SDS-PAGE using NuPAGE^® ^4-12% Bis-Tris Gel, NuPAGE MES SDS running buffer (Invitrogen Carlsbad, CA) and visualized by Coomassie staining (Bio-Rad, Hercules, CA).

### Restoration of complement classical pathway

The activity of purified rhC2 was determined using the Total Complement (3-pathway) ELISA (Alpco, Salem, NH) according to manufacturer instructions. Pooled human serum that had been stripped of C2 by affinity purification (EMD BioSciences, San Diego, CA) was reconstituted with various concentrations of rhC2 and tested. The functional activity of rhC2 was also analyzed by hemolysis in gel assays that detect deficiencies in the classical and alternative pathways of complement activation as well as deficiencies in the terminal sequence. In the classical pathway assay antibody-coated sheep red blood cells were suspended in 0.6% agarose (SeaKem^R ^ME agarose, Cambrex BioScience Rockland Inc., Rockland, ME) in the presence of Ca^2+ ^and Mg^2+^. Guinea pig erythrocytes in the presence of Mg^2+ ^and EGTA were used similarly in the assay for the alternative pathway. Samples were added to holes punched in the agarose in 5 μl volumes and allowed to diffuse into the agar in moist atmosphere at 4°C for 18 hours and then at 37°C for 2 hours for the lytic reaction to proceed [18].

### Deposition of C3 fragments on bacteria

*Streptococcus pneumoniae *23F, obtained from Statens Serum Institut (Copenhagen, Denmark) were cultured on blood agar plates and then grown in Todd-Hewitt broth until log-phase was reached. The bacteria were harvested, washed in 0.15 M NaCl and killed by adding 0.8% glutaraldehyde in 0.15 M NaCl for 20 min. The killed bacteria were stored in small aliquots at -80°C until use. To measure C3 deposition on the bacteria, serum (100 μl), diluted 1/20 in VBSCaMg, was added to 2.5 μl bacteria and incubated for 30 min at 37°C with continuous agitation. The incubation was stopped by adding 500 μl of ice-cold VBSCaMg. The bacteria were washed (centrifugation 2,880 × g, 15 min) and then 100 μl of anti-C3d (Quidel, Santa Clara, CA), diluted 1/100 in VBSCaMg was added followed by an incubation at 4°C for 30 min. After the incubation the bacteria were washed and resuspended in 100 μl VBSCaMg and 5 μl FITC-conjugated polyclonal F(ab')2 rabbit anti-mouse immunoglobulins (DakoCytomation, Glostrup, Denmark) was added. The tubes were then incubated for 30 min at 4°C. Prior to analysis by flow cytometry (Epics XL-MCL Beckman-Coulter, Fullerton, CA), the bacteria were washed once and resuspended in 500 μl VBSCaMg.

### C2-deficient patients

Sera from 6 patients with homozygous C2 deficiency were used. Studies on the C2-deficient patients were approved by the Lund University Research Ethics Committee (LU 513-01) and written informed consent was obtained for each patient. The 6 individuals all had type I C2 deficiency as ascertained by DNA analysis, and had been reported earlier [7]. MBL genotypes had also been determined as reported [26]. Only one of these patients was homozygous for the wild-type variant of the structural MBL2 gene giving rise to high concentration of MBL and lectin pathway activity. The others were heterozygous for this allele and depending on promoter variants they showed lower concentrations of serum MBL to varying degrees.

## Abbreviations

SLE: systemic lupus erythematosus; C2: complement component C2; rhC2: recombinant human complement component C2; MBL: mannose-binding lectin; MASP: MBL-associated serine protease; MAC: membrane attack complex; MALS: multi angle static light scattering; SEC: size exclusion chromatography; DSC: differential light scattering; PNGase-F: Peptide N-glycosidase-F; MFI: mean fluorescence index.

## Authors' contributions

PGVM wrote the manuscript, AOT, PGVM, LT, JLP, MWH, MFC conceived and designed the experiments, LCC, SA, CB, JG, KJZ, NS, JL produced rhC2 and tested in-vitro, AWN, DML, SMF, KL purified and analyzed rhC2, GJ, CL, BG, LT provided patients serum and performed experiments. All the authors read and approved the final manuscript.

## References

[B1] Botto M Kirschfink M Macor P Pickering MC Wurzner R Tedesco F Complement in human diseases: Lessons from complement deficiencies Mol Immunol 2009 46 2774 83 10.1016/j.molimm.2009.04.029 19481265

[B2] Figueroa JE Densen P Infectious diseases associated with complement deficiencies Clin Microbiol Rev 1991 4 359 95 1889047 10.1128/cmr.4.3.359 PMC358203

[B3] Pickering MC Botto M Taylor PR Lachmann PJ Walport MJ Systemic lupus erythematosus, complement deficiency, and apoptosis Adv. Immunol 2001 76 227 324 full_text 10.1016/s0065-2776(01)76021-x 11079100

[B4] Walport MJ Complement. First of two parts N Engl J Med 2001 344 1058 66 10.1056/NEJM200104053441406 11287977

[B5] Wallis R Dodds AW Mitchell DA Sim RB Reid KB Schwaeble WJ Molecular interactions between MASP-2, C4, and C2 and their activation fragments leading to complement activation via the lectin pathway J Biol Chem 2007 282 7844 51 10.1074/jbc.M606326200 17204478

[B6] Tedesco F Inherited complement deficiencies and bacterial infections Vaccine 2008 26 Suppl 8 I3 8 10.1016/j.vaccine.2008.11.010 19388157

[B7] Jonsson G Truedsson L Sturfelt G Oxelius VA Braconier JH Sjoholm AG Hereditary C2 deficiency in Sweden: frequent occurrence of invasive infection, atherosclerosis, and rheumatic disease Medicine (Baltimore) 2005 84 23 34 10.1097/01.md.0000152371.22747.1e 15643297

[B8] Truedsson L Alper CA Awdeh ZL Johansen P Sjoholm AG Sturfelt G Characterization of type I complement C2 deficiency MHC haplotypes. Strong conservation of the complotype/HLA-B-region and absence of disease association due to linked class II genes J Immunol 1993 151 5856 63 7901282

[B9] Wetsel RA Kulics J M-L Lokki Kiepiela P Akama H Johnson CAC Densen P Colten HR Type II human complement C2 deficiency. Allele-specific amino acid substitutions (Ser189 - > Phe; Gly444 - > Arg) cause impaired C2 secretion J. Biol. Chem 1996 271 5824 31 10.1074/jbc.271.10.5824 8621452

[B10] Barilla-LaBarca ML Atkinson JP Rheumatic syndromes associated with complement deficiency Curr Opin Rheumatol 2003 15 55 60 10.1097/00002281-200301000-00010 12496511

[B11] Ruddy S Klemperer MR Rosen FS Austen KF Kumate J Hereditary deficiency of the second component of complement (C2) in man: correlation of C2 haemolytic activity with immunochemical measurements of C2 protein Immunology 1970 18 943 54 4987909 PMC1455728

[B12] Hudson-Peacock MJ Joseph SA Cox J Munro CS Simpson NB Systemic lupus erythematosus complicating complement type 2 deficiency: successful treatment with fresh frozen plasma Br J Dermatol 1997 136 388 92 10.1111/j.1365-2133.1997.tb14951.x 9115923

[B13] Steinsson K Erlendsson K Valdimarsson H Successful plasma infusion treatment of a patient with C2 deficiency and systemic lupus erythematosus: clinical experience over forty-five months Arthritis Rheum 1989 32 906 13 2751722

[B14] Kerr MA Porter RR The purification and properties of the second component of human complement Biochem J 1978 171 99 107 417728 10.1042/bj1710099 PMC1184138

[B15] Nagasawa S Stroud RM Cleavage of C2 by C1s into the antigenically distinct fragments C2a and C2b: demonstration of binding of C2b to C4b Proc Natl Acad Sci USA 1977 74 2998 3001 10.1073/pnas.74.7.2998 70787 PMC431380

[B16] Bentley DR Primary structure of human complement component C2. Homology to two unrelated protein families Biochem J 1986 239 339 45 2949737 10.1042/bj2390339 PMC1147286

[B17] Bork K Horstkorte R Weidemann W Increasing the sialylation of therapeutic glycoproteins: the potential of the sialic acid biosynthetic pathway J Pharm Sci 2009 98 3499 508 10.1002/jps.21684 19199295

[B18] Truedsson L Sjoholm AG Laurell AB Screening for deficiencies in the classical and alternative pathways of complement by hemolysis in gel Acta Pathol Microbiol Scand [C] 1981 89 161 6 7032205 10.1111/j.1699-0463.1981.tb02680.x

[B19] Davies KA Erlendsson K Beynon HL Peters AM Steinsson K Valdimarsson H Walport MJ Splenic uptake of immune complexes in man is complement-dependent J Immunol 1993 151 3866 73 8376807

[B20] Fukuoka Y Seino J Okuda T Tachibana T Purification of the fourth, second and fifth components of mouse complement Immunology 1984 51 493 501 6698577 PMC1454464

[B21] Schultz DR Arnold PI Separation of functionally or highly pure C2 from human plasma with Sepharose and a lectin of Euonymus europeus Acta Pathol Microbiol Immunol Scand Suppl 1984 284 59 66 6587744

[B22] Thielens NM Villiers MB Reboul A Villiers CL Colomb MG Human complement subcomponent C2: purification and proteolytic cleavage in fluid phase by C1s, C1r2-C1s2 and C1 FEBS Lett 1982 141 19 24 10.1016/0014-5793(82)80006-9 6282646

[B23] Wagner E Platt JL Howell DN Marsh HC Jr Frank MM IgG and complement-mediated tissue damage in the absence of C2: evidence of a functionally active C2-bypass pathway in a guinea pig model J Immunol 1999 163 3549 58 10477630

[B24] Erlendsson K Traustadottir K Freysdottir J Steinsson K Jonsdottir I Valdimarsson H Reciprocal changes in complement activity and immune-complex levels during plasma infusion in a C2-deficient SLE patient Lupus 1993 2 161 5 10.1177/096120339300200306 8369807

[B25] Alper CA Xu J Cosmopoulos K Dolinski B Stein R Uko G Larsen CE Dubey DP Densen P Truedsson L Immunoglobulin deficiencies and susceptibility to infection among homozygotes and heterozygotes for C2 deficiency J Clin Immunol 2003 23 297 305 10.1023/A:1024540917593 12959222

[B26] Jonsson G Oxelius VA Truedsson L Braconier JH Sturfelt G Sjoholm AG Homozygosity for the IgG2 subclass allotype G2M(n) protects against severe infection in hereditary C2 deficiency J Immunol 2006 177 722 8 16785571 10.4049/jimmunol.177.1.722

[B27] Yuste J Sen A Truedsson L Jonsson G Tay LS Hyams C Baxendale HE Goldblatt F Botto M Brown JS Impaired opsonization with C3b and phagocytosis of Streptococcus pneumoniae in sera from subjects with defects in the classical complement pathway Infect Immun 2008 76 3761 70 10.1128/IAI.00291-08 18541650 PMC2493217

[B28] Neufeld EF Platt FMaW Enzyme Replacement Therapy. Lysosomal Disorders of the Brain 2004 S.V Oxford University Press 327 338

[B29] Kreuz W Martinez-Saguer I Aygoren-Pursun E Rusicke E Heller C Klingebiel T C1-inhibitor concentrate for individual replacement therapy in patients with severe hereditary angioedema refractory to danazol prophylaxis Transfusion 2009 49 1987 95 10.1111/j.1537-2995.2009.02230.x 19497056

[B30] Cugno M Zanichelli A Foieni F Caccia S Cicardi M C1-inhibitor deficiency and angioedema: molecular mechanisms and clinical progress Trends Mol Med 2009 15 69 78 10.1016/j.molmed.2008.12.001 19162547

[B31] Valdimarsson H Infusion of plasma-derived mannan-binding lectin (MBL) into MBL-deficient humans Biochem Soc Trans 2003 31 768 9 10.1042/BST0310768 12887300

[B32] Petersen KA Matthiesen F Agger T Kongerslev L Thiel S Cornelissen K Axelsen M Phase I safety, tolerability, and pharmacokinetic study of recombinant human mannan-binding lectin J Clin Immunol 2006 26 465 75 10.1007/s10875-006-9037-z 16990992

[B33] Tabor E Epstein JS NAT screening of blood and plasma donations: evolution of technology and regulatory policy Transfusion 2002 42 1230 7 10.1046/j.1537-2995.2002.00183.x 12430684

[B34] Arlaud GJ Sim RB Duplaa AM Colomb MG Differential elution of Clq, Clr and Cls from human Cl bound to immune aggregates. Use in the rapid purification of Cl subcomponents Mol Immunol 1979 16 445 50 10.1016/0161-5890(79)90069-5 40870

